# Independent Low-Rank Matrix Analysis-Based Automatic Artifact Reduction Technique Applied to Three BCI Paradigms

**DOI:** 10.3389/fnhum.2020.00173

**Published:** 2020-06-09

**Authors:** Suguru Kanoga, Takayuki Hoshino, Hideki Asoh

**Affiliations:** ^1^Artificial Intelligence Research Center, Department of Information Technology and Human Factors, National Institute of Advanced Industrial Science and Technology (AIST), Tokyo, Japan; ^2^Graduate School of Media and Governance, Keio University, Kanagawa, Japan

**Keywords:** electroencephalogram, brain–computer interface, independent component analysis, artifact reduction, independent low-rank matrix analysis

## Abstract

Electroencephalogram (EEG)-based brain-computer interfaces (BCIs) can potentially enable people to non-invasively and directly communicate with others using brain activities. Artifacts generated from body activities (e.g., eyeblinks and teeth clenches) often contaminate EEGs and make EEG-based classification/identification hard. Although independent component analysis (ICA) is the gold-standard technique for attenuating the effects of such contamination, the estimated independent components are still mixed with artifactual and neuronal information because ICA relies only on the independence assumption. The same problem occurs when using independent vector analysis (IVA), an extended ICA method. To solve this problem, we designed an independent low-rank matrix analysis (ILRMA)-based automatic artifact reduction technique that clearly models sources from observations under the independence assumption and a low-rank nature in the frequency domain. For automatic artifact reduction, we combined the signal separation technique with an independent component classifier for EEGs named ICLabel. To assess the comparative efficiency of the proposed method, the discriminabilities of artifact-reduced EEGs using ICA, IVA, and ILRMA were determined using an open-access EEG dataset named OpenBMI, which contains EEG data obtained through three BCI paradigms [motor-imagery (MI), event-related potential (ERP), and steady-state visual evoked potential (SSVEP)]. BCI performances were obtained using these three paradigms after applying artifact reduction techniques, and the results suggested that our proposed method has the potential to achieve higher discriminability than ICA and IVA for BCIs. In addition, artifact reduction using the ILRMA approach clearly improved (by over 70%) the averaged BCI performances using artifact-reduced data sufficiently for most needs of the BCI community. The extension of ICA families to supervised separation that leaves the discriminative ability would further improve the usability of BCIs for real-life environments in which artifacts frequently contaminate EEGs.

## 1. Introduction

An electroencephalogram (EEG)-based brain–computer interface (BCI) is a well-established technology that enables communicating with others without performing actual body movements by finding specific brain activity patterns from EEGs and converting these into predefined commands (Wolpaw et al., 2002). Several paradigms are used for eliciting robust time-independent or -dependent potentials in EEGs, such as motor imagery (MI) (Pfurtscheller and Da Silva, 1999), event-related potential (ERP) (Squires et al., 1976), and steady-state visual evoked potential (SSVEP) (Regan, 1966). Along with these paradigms, developments in machine-learning-based classifiers/identifiers have contributed to improvements in finding specific (elicited) patterns. These paradigms, in combination with signal processing modules and emerging technologies (e.g., wearable sensing, mobile computing, and virtual/augmented reality), have attracted increasing attention in many domains, such as medicine and robotics for real-world applications (Wang et al., 2018; Ogino et al., 2019; Vourvopoulos et al., 2019).

Artifact potentials must be reduced in all EEGs in order to realize robust real-world BCI applications because strong artifact contamination effects can easily reduce BCI performances (Kanoga et al., 2019b). During EEG measurements in real environments, biological artifacts like muscular and ocular ones cannot be avoided because it is difficult for people to voluntarily control the number of their artifact-generating activities. For example, healthy adult males blink ~20 times per minute (i.e., once every ~3 s) to maintain the moisture of their eyes (Karson, 1983). Most BCI paradigms provide visual stimuli or cues for eliciting specific neuronal patterns, and the abovementioned artifacts could contaminate the resulting EEGs. Further, muscular and ocular artifacts will contaminate all EEGs as long as the scalp has some conductivity. These unavoidable artifacts have high-amplitude electrical potentials and overlapping frequency characteristics compared to EEGs (Halliday et al., 1998; Hagemann and Naumann, 2001); thus, contamination by such artifacts makes EEG-based classification/identification hard. These contamination effects can be attenuated by increasing the distance from the source (Kanoga et al., 2016).

For denoising the contamination effects in EEG analysis, the well-known and powerful blind source separation (BSS) technique based on independent component analysis (ICA) has been widely used for the last 20 years (Jung et al., 2000). Usually, artifact reduction involves three steps: (1) training a demixing matrix, (2) identifying the types of separated independent components (ICs), and (3) remixing EEGs by using only neuronal ICs and an inverse demixing matrix. To improve both the computational cost and the accuracy of training a demixing matrix, many ICA algorithms, such as fast ICA (Hyvärinen and Oja, 1997), second-order blind interference (SOBI) (Belouchrani et al., 1997), and information maximization (infomax) ICA (Bell and Sejnowski, 1995), have been proposed. The SOBI and infomax ICA algorithms are used most commonly for EEG signal processing (Choi et al., 2005; Urigüen and Garcia-Zapirain, 2015). For example, EEGLAB, an enormous interactive toolbox for EEG analysis, implements the infomax ICA algorithm (Delorme and Makeig, 2004). Recently, ICLabel (Pion-Tonachini et al., 2019), an automatic IC classifier, has been integrated into the EEGLAB toolbox for online streaming EEG data. Overall, an ICA-based approach remains the gold-standard for artifact reduction.

While real-world EEG-based BCI applications are being developed steadily, ICA-based source estimation still poses some problems. ICA algorithms comprehensively minimize the reconstruction error with a linear combination for an entire sequence of trials. However, this approach overestimates sources for representing the latent waveform of an observation; thus, the estimation leads to oversubtraction or spectral distortion of the EEG power (Wallstrom et al., 2004; Castellanos and Makarov, 2006). Proposing a more rigorous representation of estimated sources than ICA is a major challenge in EEG signal processing for constructing effective classifiers/identifiers.

To represent meaningful waveforms from an observation, we focused on the recurrent properties of an artifactual waveform over trials. Biological artifacts are based on a person's organ structure. An organ system reproducibly and unconsciously activates a non-cerebral source (e.g., eyeball) in the same manner and generates similar electrical potentials (e.g., electrooculogram signals); therefore, person-specific artifacts share a few basic functions for representing the waveforms and can be considered low-rank matrices comprising multiple short time segments. This study represents and removes such waveforms using an independent low-rank matrix analysis (ILRMA) that finds a low-rank non-negative matrix based on statistical independence (Kitamura et al., 2016). To improve its usability for EEG analysis, we used ICLabel for artifact reduction. We investigated the discriminabilities of artifact-reduced EEGs obtained by different methods by using OpenBMI, an open-access EEG dataset that contains EEG data, through the three abovementioned BCI paradigms (MI, ERP, and SSVEP). The BCI performances with these three paradigms after applying artifact reduction techniques were obtained. The results suggest that the proposed method can potentially achieve higher discriminability than ICA for BCIs.

## 2. Artifact Reduction Techniques

### 2.1. Mixing and Demixing of EEGs

A typical approach to artifact reduction in EEG observations is based on the following assumption: *P*-channel EEG observations are overdetermined/determined and linear combinations of unknown cerebral *Q* sources including artifactual and neuronal ones and white noises. Neuronal cells have limited propagation because the cortical connectivity is highly weighted toward short (<500 μm) connections (Budd and Kisvárday, 2001). Thus, the electrical potentials of neuronal activities spread through a contiguous cortical region with a high attenuation penalty in proportion with the distance from the sources (Arieli et al., 1996; Onton and Makeig, 2006). By matching the underlying dynamics of the generation and propagation of EEG potentials, the aforementioned assumption can be represented as

(1) $$\begin{array}{r} x\left(n\right)=As\left(n\right)+d\left(n\right), \end{array}$$

where $x\left(n\right)\;=\;{\left[x_{1}\left(n\right),x_{2}\left(n\right),\ldots ,x_{P}\left(n\right)\right]}^{\text{T}}$ is the EEG observation at the *n*th sampling point (1 ≤ *n* ≤ *N*), $s\left(n\right)\;=\;{\left[s_{1}\left(n\right),s_{2}\left(n\right),\ldots ,s_{Q}\left(n\right)\right]}^{\text{T}}$ is the unknown source, ***A*** is the *P* × *Q* full-rank unknown mixing matrix, and $d\left(n\right)\;=\;{\left[d_{1}\left(n\right),d_{2}\left(n\right),\ldots ,d_{P}\left(n\right)\right]}^{\text{T}}$ is the additive zero-mean noise (^T^ indicates the transpose).

Here, we have only an artifact-(un)contaminated EEG observation matrix ***X*** = [***x***(1), …, ***x***(*N*)] ∈ ℝ^*P*×*N*^. Because source estimation through the inverse process is evidently intractable, four additional assumptions are made (James and Hesse, 2004; Vigario and Oja, 2008): (1) the noise is spatially uncorrelated with the observed data (𝔼[***As***(*n*)***d***(*n*)] = **0**, where 𝔼[·] is the expectation operator); (2) the noise is temporally uncorrelated (𝔼[***d***(*n*)***d***(*n* + τ)] = **0**, where τ is the lag time (∀τ > 0)); (3) the number of sources is equal to or lower than the number of observations (*P* ≥ *Q*); and (4) the mixing matrix ***A*** does not change over time. Under these assumptions, we can simultaneously estimate both the source matrix $\hat{S}=\left[\hat{s}\left(1\right),\ldots ,\hat{s}\left(N\right)\right]\in {\mathbb{R}}^{Q\times N}$ and the demixing matrix ***W***(= ***A***^−1^) ∈ ℝ^*Q*×*P*^ to blindly separate the observations into artifactual/neuronal sources:

(2) $$\begin{array}{r} \hat{s}\left(n\right)=Wx\left(n\right). \end{array}$$

**Figure 1:**
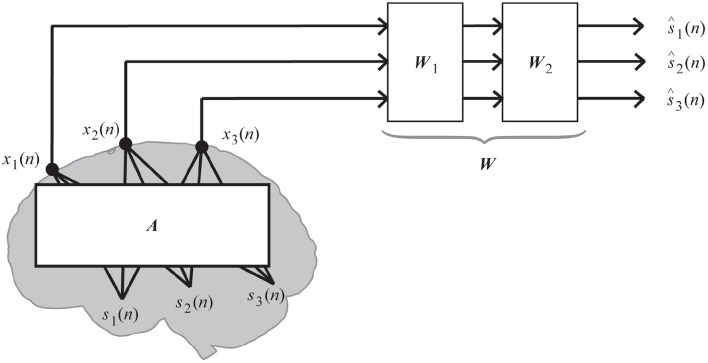
Linear mixing and demixing of EEGs (Kanoga and Mitsukura, 2017).

The linear mixing and demixing of EEGs shown in Figure 1 accounts for the comprehensive demixing matrix ***W***(= ***W***_1_***W***_2_) because signal separation algorithms sometimes first decorrelate the data by ***W***_1_ and then demix them by ***W***_2_, which is originally learned from the algorithm. In this study, we decorrelated the data before applying a matrix factorization technique; thus, the following representation of ***W*** has the same meaning as ***W***_2_ in Figure 1.

In practice, artifact reduction requires three stages of processing: (1) decomposing the input matrix; (2) identifying whether the decomposed component is artifactual or neuronal; and (3) reconstructing the artifact-reduced signals using only neuronal components. In this case, we assumed that the EEG observations are labeled signals; the dimensionality of the sources and observations is the same (the value of *P* and *Q* is 20, 32, or 10 for MI, ERP, or SSVEP paradigm. The value depends on the number of selected channels in the BCI paradigm. More detailed information is described in sections 3.1, 3.2, and 3.3). In addition, we decomposed datasets using three BSS methods for ICA families: ICA, independent vector analysis (IVA), and ILRMA. Then, the decomposed components were automatically identified using the ICLabel function in the EEGLAB toolbox proposed by Pion-Tonachini et al. (2019). Based on the labels, artifact-reduced signals were linearly reconstructed.

Although ICA algorithms handle time-series data, IVA and ILRMA algorithms approximate a bin-wise instantaneous mixture model in a short-time Fourier transform (STFT) domain. An EEG time series is transformed into a sequence of complex-valued signals by using STFT with a 50% overlapped 1-s Hamming window. Thus, the observations and sources in each time-frequency slot are described as $x_{ij}={\left[x_{ij1},x_{ij2},\ldots ,x_{ijP}\right]}^{\text{T}}\in {\mathbb{C}}^{P}$ and ${\hat{s}}_{ij}={\left[{ŝ}_{ij1},{ŝ}_{ij2},\ldots ,{ŝ}_{ijQ}\right]}^{\text{T}}\in {\mathbb{C}}^{Q}$, where a couple of (*i, j*) defines the *i*th frequency bin and *j*th time frame over STFT (1 ≤ *i* ≤ *I* and 1 ≤ *j* ≤ *J*). IVA and ILRMA algorithms assume the following mixing system:

(3) $$\begin{array}{r} x_{ij}=A_{i}s_{ij}, \end{array}$$

where $A_{i}={\left[a_{i1},a_{i2},\ldots ,a_{iQ}\right]}^{\text{H}}\in {\mathbb{C}}^{P\times Q}$ is a frequency-wise mixing matrix (***a***_*iq*_ is the steering vector for the *q*th source, and ^H^ indicates the Hermitian transpose). In this paper, we set the value of time length to 1 s because some frequency-domain artifact reduction techniques translate EEG data into STFT domain based on 1-s windows (Kanoga and Mitsukura, 2014; Mohammadpour and Rahmani, 2017) and ILRMA showed its high separation accuracy for 1-s time length data (Kitamura et al., 2017). This mixing system is the rank-1 spatial model (Duong et al., 2010); thus, the relationships between observations and sources can be represented:

(4) $$\begin{array}{r} {\hat{s}}_{ij}\approx y_{ij}=W_{i}x_{ij}, \end{array}$$

where $y_{ij}={\left[y_{ij1},y_{ij2},\ldots ,y_{ijQ}\right]}^{\text{T}}\in {\mathbb{C}}^{Q}$ is the STFT of the estimated signals and $W_{i}={\left[w_{i1},w_{i2},\ldots ,w_{iQ}\right]}^{\text{H}}={A_{i}}^{-1}\in {\mathbb{C}}^{Q\times P}$ is the demixing matrix. Note that the demixing matrices for IVA, ILRMA, and ICA have different dimensionalities because of the differences in the domain used (IVA and ILRMA: ***W*** ∈ ℝ^*Q*×*P*×*I*^, ICA: ***W*** ∈ ℝ^*Q*×*P*^).

### 2.2. Matrix Factorization Techniques

#### 2.2.1. Independent Component Analysis

ICA is the most famous classical method for separating multichannel EEG observations ***x***(*n*) into statistically independent sources $\hat{s}\left(n\right)$ based on an estimated demixing matrix ***W*** (Jung et al., 2000; Delorme et al., 2007). The sources can be said to be statistically independent when the following relationship holds:

(5) $$\begin{array}{r} p\left(\hat{s}\right)=\overset{Q}{\underset{q=1}{\prod }}p\left({\hat{s}}_{q}\right), \end{array}$$

where $p\left(\hat{s}\right)$ and $p\left({\hat{s}}_{q}\right)$ are the joint and the marginal probability distribution of the sources, respectively. Thus, ICA algorithms optimize the demixing matrix ***W*** by minimizing the dependence between these distributions. This study applied the extended infomax ICA algorithm implemented by Lee et al. (1999) using the runica function in EEGLAB to the observations. In this algorithm, the dependence in the distributions is represented as the mutual information (Kullback–Leibler distribution) between the estimated sources and observations $I\left(\hat{s};x\right)$:

(6) $$\begin{array}{r} I\left(\hat{s};x\right)=H\left(\hat{s}\right)-\overset{Q}{\underset{q=1}{\sum }}H\left({\hat{s}}_{q}\right), \end{array}$$

where

(7) $$\begin{array}{r} H\left(\hat{s}\right)=-\int p\left(\hat{s}\right)\log p\left(\hat{s}\right)d\hat{s}, \end{array}$$

(8) $$\begin{array}{r} H\left({\hat{s}}_{q}\right)=-\int p\left({\hat{s}}_{q}\right)\log p\left({\hat{s}}_{q}\right)d{\hat{s}}_{q}. \end{array}$$

By applying the relationship $p\left(x\right)=p\left(\hat{s}\right)/|\det W|$ to Equation (7), Equation (6) can be rewritten as a cost function for optimizing the demixing matrix:

(9) $$\begin{array}{r} I\left(W\right)=\text{const}.-\overset{Q}{\underset{q=1}{\sum }}H\left({\hat{s}}_{q}\right)-\log|\det W|. \end{array}$$

The entropy of given observations H(x) is a constant. In addition, a gradient update rule based on the natural gradient (Amari, 1998) with learning rate η is used to solve the optimization problem:

(10) $$\begin{array}{r} W\leftarrow W+\eta \Delta W, \end{array}$$

where

(11) $$\begin{array}{r} \Delta W=\left(I-𝔼\left[\varphi \left(\hat{s}\right){\hat{s}}^{T}\right]\right)W. \end{array}$$

In every iteration, the distribution of the estimated source for the score function $\varphi \left({\hat{s}}_{q}\right)$ is chosen from the super-Gaussian or sub-Gaussian based on the sign of the fourth cumulant of each source $c_{4}=M_{4}-3M_{2}^{2}$, where *M*_*k*_ is the *k*th moment ($M_{k}=𝔼\left[{\hat{s}}_{q}^{k}\right]$).

(12) $$\begin{array}{r} \varphi \left({\hat{s}}_{q}\right)=-\left({\hat{s}}_{q}+\text{sgn}\left(c_{4}\right)\text{tanh}\left({\hat{s}}_{q}\right)\right). \end{array}$$

In a real environment, the expectation operator 𝔼[·] is the expected value of the empirical distribution (the sample average of the variable).

#### 2.2.2. Independent Vector Analysis

IVA is an extension of the ICA algorithm to multivariate components (vectorized signals) (Hiroe, 2006; Kim et al., 2006b). Like ICA algorithms, IVA defines the dependence between joint probability distributions and marginal probability products using the Kullback–Leibler divergence; however, it introduces a vector density model that has a variance dependency within a source vector. This study applied the natural-gradient-based IVA algorithm implemented by Kim et al. (2006b) based on the ivabss function from an open-access toolbox available on Github (https://github.com/teradepth/iva).

Two conditions are assumed: (1) elements of a source vector are mutually independent of those of other source vectors; and (2) within a source vector, the elements are highly dependent on each other. Based on these assumptions, the cost function for multivariate random variables to separate the components from the observations can be written as

(13) $$\begin{array}{r} I\left(W\right)=\text{const}.-\overset{Q}{\underset{q=1}{\sum }}H\left({\hat{s}}_{q}\right)-\overset{I}{\underset{i=1}{\sum }}\log|\det W_{i}|. \end{array}$$

The cost function preserves the inherent dependency within each source vector, though it removes the dependency between different source vectors.

By differentiating the object function with respect to the coefficients of demixing matrices ***W***_*i*_ and using the natural gradient, we can derive a gradient update rule as

(14) $$\begin{array}{r} \Delta W_{i}\leftarrow W_{i}+\eta \Delta W_{i}, \end{array}$$

where

(15) $$\begin{array}{r} \Delta W_{i}=\left(I-𝔼\left[\varphi_{i}\left({\hat{s}}_{1},\ldots ,{\hat{s}}_{I}\right){\hat{s}}_{i}^{⋆}\right)\right]W_{i}, \end{array}$$

where ***a***^⋆^ denotes the complex conjugate of ***a***. Among a number of possible function forms, one of the simplest and most effective score functions is given as follows:

(16) $$\begin{array}{r} \varphi_{i}\left({\hat{s}}_{1},\ldots ,{\hat{s}}_{I}\right)=\frac{{\hat{s}}_{i}}{\sqrt{\sum_{i=1}^{I}{\left|{\hat{s}}_{i}\right|}^{2}}} \end{array}$$

To define an optimal form of the function $p\left({\hat{s}}_{q}\right)$, which has dependency within a source vector, IVA algorithms introduce a vector density model as a scale mixture of a Gaussian distribution with a fixed mean and a variable variance (Kim et al., 2006a):

(17) $$\begin{array}{r} p\left({\hat{s}}_{q}\right)=\overset{I}{\underset{i=1}{\prod }}p\left({\hat{s}}_{iq}\right)=\alpha \overset{I}{\underset{i=1}{\prod }}\text{exp}\left(-\sqrt{{\left({\hat{s}}_{iq}-\mu_{iq}\right)}^{†}{\Sigma_{iq}}^{-1}\left({\hat{s}}_{iq}-\mu_{iq}\right)}\right) \end{array}$$

where α is a normalization term, μ_*iq*_ and Σ_*iq*_ are respectively the mean vector and covariance matrix of the *q*th source signal in the *i*th frequency bin, and ***a***^†^ is the conjugate transpose of ***a***.

#### 2.2.3. Independent Low-Rank Matrix Analysis

The ILRMA method unifies IVA and non-negative matrix factorization (Lee and Seung, 1999; Sawada et al., 2013) by considering the determined situation (*P* = *Q*) and a linear time-invariant mixing system (Kitamura et al., 2016). This study applied the ILRMA algorithm implemented by Kitamura et al. (2016) using the ILRMA function from an open-access toolbox available on Github (https://github.com/d-kitamura/ILRMA). The algorithm estimates both the demixing matrix ***W***_*i*_ and the STFT of the estimated signals ***y***_*ij*_ by approximately decomposing $|y_{ijq}{|}^{2}$ into the non-negative elements *t*_*ik*_ and *v*_*kj*_ of the basis matrix $T_{q}\in {\mathbb{R}}^{I\times K}$ and the activation matrix $V_{q}\in {\mathbb{R}}^{K\times J}$ with a latent variable *z*_*qk*_ of the partitioning function ***Z***, which indicates whether or not the *k*th basis (1 ≤ *k* ≤ *K*) belongs to the *q*th source. For the decomposition, the ILRMA algorithm has the following cost function:

(18) $$\begin{array}{r} I\left(W\right)=\underset{ij}{\sum }\left\{\underset{q}{\sum }\log\underset{k}{\sum }z_{qk}t_{ik}v_{kj}+\underset{q}{\sum }\frac{{\left|y_{ijq}\right|}^{2}}{\sum_{k}z_{qk}t_{ik}v_{kj}}-2\log|\det W_{i}|\right\}, \end{array}$$

where $y_{ijq}={w_{iq}}^{\text{H}}x_{ij}$. The cost function finds a low-rank time-frequency structure for sources using the first and second terms in Equation (18) and maximizes the statistical independence between sources using the second and third terms in Equation (18).

In this algorithm, the demixing matrix ***W***_*i*_ can be efficiently updated through iterative projection based on the auxiliary function technique (Ono, 2011):

(19) $$\begin{array}{r} V_{iq}=\frac{1}{J}\underset{j}{\sum }\frac{1}{r_{ijq}}x_{ij}x_{ij}^{\text{H}}, \end{array}$$

(20) $$\begin{array}{r} w_{iq}\leftarrow {\left(W_{i}V_{iq}\right)}^{-1}e_{q}, \end{array}$$

(21) $$\begin{array}{r} w_{iq}\leftarrow w_{iq}{\left(w_{iq}^{H}V_{iq}w_{iq}\right)}^{-\frac{1}{2}}, \end{array}$$

where *r*_*ijq*_ is the estimated variance of each source under the complex Gaussian distribution and ***e***_*q*_, a unit vector in which the *q*th element is equal to unity. These update rules have been reported to be faster and more stable than conventional update rules (e.g., natural gradient). After the update, the separated signal ***y***_*ij*_ is also updated:

(22) $$\begin{array}{r} y_{ijq}\leftarrow w_{iq}^{\text{H}}x_{ij}. \end{array}$$

In addition, the basis matrix ***T***_*q*_, activation matrix ***V***_*q*_, and partitioning function ***Z*** can be updated by the majorization-minimization algorithm (Hunter and Lange, 2000):

(23) $$\begin{array}{r} z_{qk}\leftarrow z_{qk}\sqrt{\frac{\sum_{ij}|y_{ijq}{|}^{2}t_{ik}v_{kj}{\left(\sum_{k^{\prime }}z_{qk^{\prime }}t_{ik^{\prime }}v_{k^{\prime }j}\right)}^{-2}}{\sum_{ij}t_{ik}v_{kj}{\left(\sum_{k^{\prime }}z_{qk^{\prime }}t_{ik^{\prime }}v_{k^{\prime }j}\right)}^{-1}}}, \end{array}$$

(24) $$\begin{array}{r} t_{ik}\leftarrow t_{ik}\sqrt{\frac{\sum_{jq}|y_{ijq}{|}^{2}z_{qk}v_{kj}{\left(\sum_{k^{\prime }}z_{qk^{\prime }}t_{ik^{\prime }}v_{k^{\prime }j}\right)}^{-2}}{\sum_{jq}z_{qk}v_{kj}{\left(\sum_{k^{\prime }}z_{qk^{\prime }}t_{ik^{\prime }}v_{k^{\prime }j}\right)}^{-1}}}, \end{array}$$

(25) $$\begin{array}{r} v_{kj}\leftarrow v_{kj}\sqrt{\frac{\sum_{iq}|y_{ijq}{|}^{2}z_{qk}t_{ik}{\left(\sum_{k^{\prime }}z_{qk^{\prime }}t_{ik^{\prime }}v_{k^{\prime }j}\right)}^{-2}}{\sum_{iq}z_{qk}t_{ik}{\left(\sum_{k^{\prime }}z_{qk^{\prime }}t_{ik^{\prime }}v_{k^{\prime }j}\right)}^{-1}}}. \end{array}$$

Finally, the estimated source model is represented as

(26) $$\begin{array}{r} r_{ijq}=\underset{k}{\sum }z_{qk}t_{ik}v_{kj}. \end{array}$$

Note that the demixing matrix ***W***_*i*_ and the estimated variance *r*_*ijq*_ are normalized at each iteration to avoid the risk of diverging as follows:

(27) $$\begin{array}{r} \lambda_{q}=\sqrt{\frac{1}{IJ}\underset{ij}{\sum }|y_{ijq}{|}^{2}}, \end{array}$$

(28) $$\begin{array}{r} w_{iq}\leftarrow w_{iq}\lambda_{q}^{-1}, \end{array}$$

(29) $$\begin{array}{r} y_{ijq}\leftarrow y_{ijq}\lambda_{q}^{-1}, \end{array}$$

(30) $$\begin{array}{r} r_{ijq}\leftarrow r_{ijq}\lambda_{q}^{-2}. \end{array}$$

The number of bases for all sources *K* and number of iterations were set to *J*/10 and 200, respectively.

### 2.3. Component Identification

For identifying the estimated ICs obtained from ICA, IVA, and ILRMA, we used the ICLabel (https://github.com/sccn/ICLabel) classifier proposed by Pion-Tonachini et al. (2019) (freely available as a package in EEGLAB; Delorme and Makeig, 2004; Delorme et al., 2011). This classifier uses three artificial neural networks (ANNs) (specifically, two “Classifier” networks and one “Generator” network): (1) a convolutional neural network (CNN) optimized by an unweighted cross-entropy loss, (2) a CNN optimized by a weighted cross-entropy loss and neuronal IC classification errors, and (3) a semi-supervised learning generative adversarial network (SSGAN) (Odena, 2016; Salimans et al., 2016). The ICLabel classifier inputs, architectures, and training paradigms are described in detail in Appendices B and E in Pion-Tonachini et al. (2019). By using these three ANNs and the three IC features, the ICLabel classifier classified unlabeled EEG ICs into seven categories: (1) brain, (2) muscle, (3) eye, (4) heart, (5) line noise, (6) channel noise, and (7) others. In this study, because of the property of the epoch identification method described in section 4, we removed ICs whose labels were “muscle” or “eye.” Currently, the ICLabel classifier has been trained using 6352 EEG recordings in storage drives collected over the past 15 years at Swartz Center for Computational Neuroscience (SCCN) at UC San Diego.

To accurately identify IC labels using the ICLabel classifier, three discriminable features were calculated from each IC: (1) 32 × 32 pixel scalp topography using the topoplot function in EEGLAB, (2) median power spectral densities (PSDs) from 1 to 50 Hz using a variation of the Welch method (Welch, 1967), and (3) the autocorrelation function. The scalp topographies and PSDs were scaled such that each had a maximum absolute value of 0.99. Further, the autocorrelation vectors were normalized such that the zero-lag value was 0.99. Note that the estimated mixing matrix (***W***^−1^), channel locations in 3D space, and channel labels were required to generate the scalp topographies. We collected information about the channel locations in 3D space from the sample_locs folder in EEGLAB toolbox. In addition, the estimated demixing matrix has a 3D structure except for ICA; thus, the corresponding frequency band (e.g., 8–30 Hz) was extracted from whole frequency bins, and the summation was computed to transform the matrix into a 2D structure. Furthermore, the matrix was scaled by the number of extracted frequency bins.

### 2.4. Signal Reconstruction

By performing the identification process using ICLabel, labels are obtained for the estimated ICs. If the label is “eye” or “muscle,” all components of the artifactual IC are set to zeros. Based on the modified sources, artifact-reduced EEG signals in EEG observations were reconstructed using the inverse linear demixing process in ICA. While applying IVA and ILRMA, the sources were translated into frequency components. Thus, all frequency components of the artifactual ICs were set to zeros and translated into time-series data by the inverse STFT. Then, the artifact-reduced EEG signals were reconstructed, and the inverse ICA linear demixing process was performed.

## 3. Materials and Baseline Methods

To assess the discriminability of artifact-reduced EEGs by ICA, IVA, and ILRMA, we downloaded an open-access EEG dataset published by Lee et al. (2019) from the webpage http://gigadb.org/dataset/view/id/100542/File_page. The EEG data were recorded using 62 electrodes according to the International 10-20 system using BrainAmp (Brain Products; Munich, Germany) with a sampling rate of 1,000 Hz. In the analysis procedures, we commonly downsampled all EEG data to 100 Hz. The reference and ground channels were nasion and AFz, respectively. The impedance of the EEG electrodes was maintained below 10 kΩ. Participants were instructed to comfortably sit in a chair with armrests ~60 cm in front of a 21-inch LCD monitor (refresh rate: 60 Hz; resolution: 1, 600 × 1, 200). In addition, they were asked to relax their muscles and minimize their eye and muscle movements during the BCI paradigms. Before beginning the experiments, five kinds of 10-s artifact-contaminated EEG data were measured: (1) eye blinking, (2) repetitive horizontal eye movements, (3) repetitive vertical eye movements, (4) teeth clenching, and (5) flexing both arms.

The dataset has the following three properties: (1) a large number of subjects (54 healthy participants; 29 males and 25 females; age: 24–35 years), (2) multiple sessions (two sessions on different days), and (3) multiple paradigms (a binary-class MI, a 36-symbol ERP, and a four-target-frequency SSVEP). Each session consisted of training and testing phases. All BCI paradigms were developed based on the OpenBMI toolbox (Lee et al., 2016) and Psychtoolbox (Brainard, 1997). We used this dataset because (1) EEGs in the three BCI paradigms were collected from the same participants, (2) each paradigm was conducted for 2 days, and (3) baseline analysis methods based on Matlab functions in the OpenBMI toolbox (https://github.com/PatternRecognition/OpenBMI) are available. A single dataset having all these properties is very important for fairly comparing algorithms to reveal general performances with intra- and inter-subject/paradigm variabilities in BCI research. To verify the change in the discrimination accuracy with artifact-reduced EEGs, the baseline analysis methods, including feature extraction and classification algorithms for each paradigm described in Lee et al. (2019), were used. Each paradigm and processing stream are described in detail in the following subsections.

### 3.1. MI Paradigm and Processing

The MI paradigm was designed based on a well-established protocol (Pfurtscheller and Neuper, 2001): a training/testing phase had 100 trials with 50 right and 50 left hand motion imagery tasks resulting in binary classification. Each trial lasted 13 ± 1.5 s. In the first 3 s, a black fixation cross appeared at the center of the monitor. After the preparation time, the participant imagined a right or left grasping motion for 4 s depending on whether a right arrow or left arrow was displayed, respectively, and then remained in the resting state for 6 ± 1.5 s.

In the MI paradigm, 20 electrodes in the motor cortex region (FC-5/3/1/2/4/6, C-5/3/1/z/2/4/6, and CP-5/3/1/z/2/4/6) were selected. The EEG data of the selected channels were band-pass filtered between 8 and 30 Hz through a fifth order Butterworth filter and further segmented into 2.5-s epochs, which are data segments of 1.0 to 3.5 s after the cue onsets (Pfurtscheller and Neuper, 2001; Fazli et al., 2009). We applied the filter bank common spatial pattern (FBCSP) to the epochs, which has been widely used in MI-based BCIs to maximize the discrimination of the binary class (Ang et al., 2008). A subset of the top and bottom two rows from the projection matrix was used for calculating log-variance features. Based on the features, linear discriminant analysis (LDA) classifiers were constructed and used.

### 3.2. ERP Paradigm and Processing

The ERP paradigm was designed based on a typical row-column speller system with random-set presentation (Yeom et al., 2014) and face stimuli (Kaufmann et al., 2011). The six rows and six columns were configured with 36 symbols (alphabets A to Z, numerals 1 to 9, and underscore “_”). Each trial sequence lasted 19.5 s. In the first 4.5 s, a target character was highlighted for attracting the participant's attention. After the preparation time, all rows and columns were flashed one by one (12 stimulus flashes) for 13 s and then remained in the resting state for 2 s. The stimulus-time interval was set to 80 ms and the interstimulus interval (ISI) to 135 ms. The highlighted target character was estimated based on data of five sequences (i.e., 60 flashes). In the training phase, participants were asked to copy the 33 characters including spaces in “NEURAL_NETWORKS_AND_DEEP_LEARNING” by gazing at the target character on the monitor, resulting in 1980 trials and binary classification (target or non-target character). In the testing phase, participants tried to copy the 36 characters including spaces in “PATTERN_RECOGNITION_MACHINE_LEARNING,” resulting in 2,160 trials.

In the ERP paradigm, 32 electrodes (Fp-1/2, F-7/3/z/4/8, FC-5/1/2/6, T-7/8, C-3/z/4, TP-9/10, CP-5/1/2/6, P-7/3/z/4/8, PO-9/10, and O-1/z/2) were selected. The EEG data of the selected channels were band-pass filtered between 0.5 and 40 Hz through a fifth order Butterworth filter and then baseline-corrected by subtracting the average amplitudes of the prestimulus within an interval of 200 ms with respect to the stimulus onset. In addition, 0.8-s epochs after the onset were extracted for analysis. From the epochs, the mean amplitudes (MAs) over eight non-overlapping samples were calculated as the 320-dimensional subject-dependent spatio-temporal features (10 dimensions, 32 channels). Based on the features, LDA classifiers were constructed and used.

### 3.3. SSVEP Paradigm and Processing

The SSVEP paradigm was designed based on general requirements for SSVEP-based BCIs that run over four specific commands (Parini et al., 2009). Four flickers at 5.45, 6.67, 8.57, and 12 Hz were displayed at four positions (down, right, left, and up) on a monitor. Each target frequency was presented 25 times for both the training and the testing phases, resulting in four target identification problems. Each trial lasted 10 s. In the first 4 s, the participant gazed in the box where the target was highlighted (not flickering) in a different color, and the target flicker was then presented for 4 s with an ISI of 6 s to induce the target SSVEP.

In the SSVEP paradigm, 10 electrodes in the occipital region (P-7/3/z/4/8, PO-9/10, and O-1/z/2) were selected. The EEG data of the selected channels were segmented into 2-s epochs with respect to the stimulus onset. We applied multichannel canonical correlation analysis (CCA) (Lin et al., 2006) for identifying the target frequency index by calculating the correlation values between the input data and the prepared sinusoidal templates of the corresponding frequencies (5.45, 6.67, 8.57, and 12 Hz). Although this identification process does not need training data owing to the use of an unsupervised classifier, only data from the testing phase were used for evaluating the BCI performance.

## 4. Assessments

In this study, we assumed that artifact-reduced epochs are correctly classified if the artifact reduction technique effectively reduced artifactual effects from artifact-contaminated epochs. However, we do not know how many epochs of the aforementioned paradigms were contaminated by artifacts because the open-access EEG dataset does not provide such information. Empirically, it is difficult to completely avoid the generation of biological artifacts during EEG paradigms. Thus, we expected that some epochs were contaminated by some artifacts during each BCI paradigm. To identify the type of epoch (not artifact-contaminated or artifact-contaminated), we applied the detection of events in continuous time (DETECT) epoch identification method proposed by Lawhern et al. (2013); this method requires training data with clean and artifactual label information to make a multiclass SVM model (https://github.com/VisLab/detect). Usually, training data has a short time length (e.g., less than 5 s). Thus, in this study, 10 1-s no artifact-contaminated EEG data detected based on a manual inspection and extracted from the training phase of each BCI paradigm as “clean” epochs and five types of 10-s EEG data contaminated by artifacts, such as eye blinking, horizontal/vertical eye movements, teeth clenching, and flexing both arms, were prepared as “artifactual” epochs because these are well-known to generate ocular/muscular artifacts during EEG measurements. For training an SVM model, each 10-s-length artifactual data was separated into 10 1-s-length data without overlapping. Based on the 60 1-s epochs (10 1-s epochs × 6 classes), a 6-class SVM model was constructed. Note that segmented epochs of the BCI paradigms have different time length (i.e., MI: 2.5, ERP: 0.8, and SSVEP: 2.0 s). To apply DETECT based on a processing strategy for 1-s-length data, we extracted first 1-s data from the “clean” epochs if the target BCI paradigm was MI or SSVEP. For the epochs of the ERP paradigm, 0.2-s data before the stimulus onsets were concatenated to the “clean” epochs. Then, autoregressive features were extracted from the epochs to construct a multiclass SVM classifier of each BCI paradigm. The classifier and hard thresholding for the estimated artifactual class (certainty value obtained using the DETECT toolbox was over 0.5 or not) finally identified each epoch as being clean or artifact contaminated. Table 1 lists the identification results. In all paradigms, data recorded in session 2 (day 2) had less artifactual data than data recorded in session 1 (day 1). In the MI and ERP paradigms, the number of artifactual data recorded in day 2 was significantly lower than data recorded in day 1 (*p* = 0.001, 0.009 for MI and ERP paradigms in *t*-test). However, in the SSVEP paradigm, the number in day 2 was not significantly lower (*p* = 0.172 in *t*-test). Note that we identify the epoch is neuronal unless a certainty value of all classes exceeded the hard threshold; thus, the thresholding process found an explicit artifactual class over 6 class labels. If the certainty values distributed throughout all classes and no one did not exceed the threshold, this modest identification method can not find artifact-contaminated epochs. In Table 1, there was an outlier: subject 5 in the session 1 of ERP paradigm had no artifact-contaminated epoch. This phenomenon might be caused by the above-mentioned reason.

**Table 1:** Number of artifact-contaminated epochs in training and testing phases of each BCI paradigm and subject.

**Subject**	**MI**				**ERP**				**SSVEP**			
	**Session 1**		**Session 2**		**Session 1**		**Session 2**		**Session 1**		**Session 2**	
	**Train**	**Test**	**Train**	**Test**	**Train**	**Test**	**Train**	**Test**	**Train**	**Test**	**Train**	**Test**
s1	6	2	8	6	149	330 (36)	80	215 (33)	3	2	0	1
s2	2	0	0	1	95	5 (5)	38	115 (25)	0	0	0	0
s3	1	0	7	10	158	62 (20)	17	14 (9)	14	10	1	2
s4	0	0	0	0	32	34 (14)	7	10 (4)	9	18	0	0
s5	4	4	7	5	0	0 (0)	395	282 (25)	19	11	14	8
s6	4	2	4	3	159	113 (24)	171	146 (29)	25	26	5	6
s7	21	30	6	8	75	200 (25)	52	21 (10)	8	7	4	4
s8	2	0	4	1	427	325 (32)	35	87 (22)	3	2	9	5
s9	0	0	9	2	43	73 (24)	145	99 (25)	1	9	0	1
s10	0	2	0	0	94	15 (6)	61	78 (20)	0	0	2	4
s11	5	4	0	0	50	56 (15)	16	47 (17)	0	0	0	0
s12	0	16	2	6	75	94 (22)	98	185 (31)	0	1	1	7
s13	1	0	1	1	131	212 (29)	139	103 (26)	6	3	3	1
s14	1	0	2	1	18	35 (11)	234	190 (32)	4	6	13	22
s15	3	20	0	1	25	19 (11)	48	7 (5)	1	1	3	16
s16	0	2	13	10	492	322 (33)	113	92 (19)	0	0	8	9
s17	27	36	4	6	232	187 (30)	263	267 (34)	1	1	15	21
s18	1	11	13	9	166	404 (31)	92	122 (29)	3	2	2	4
s19	1	8	2	7	159	300 (33)	36	169 (30)	7	15	2	3
s20	0	7	1	1	236	240 (34)	112	323 (35)	2	2	1	2
s21	31	30	0	0	69	84 (29)	268	129 (26)	15	9	3	5
s22	2	8	2	0	131	128 (32)	11	48 (18)	9	2	0	1
s23	0	0	9	0	141	194 (29)	160	227 (26)	0	36	1	1
s24	0	1	8	4	65	106 (27)	87	158 (31)	2	1	1	5
s25	3	11	6	2	184	122 (24)	14	59 (15)	2	2	8	4
s26	0	1	0	0	447	552 (36)	196	265 (32)	20	22	7	6
s27	3	12	1	1	172	312 (34)	150	346 (32)	1	5	11	14
s28	2	6	0	0	93	63 (21)	37	52 (19)	6	4	2	1
s29	8	20	2	4	32	159 (25)	101	27 (9)	3	4	3	3
s30	3	9	4	0	141	171 (34)	38	30 (14)	0	1	0	0
s31	7	3	0	1	33	82 (23)	25	60 (29)	0	0	1	0
s32	33	67	0	2	122	339 (33)	80	32 (13)	13	34	19	21
s33	0	1	4	0	119	52 (17)	107	59 (15)	0	2	1	2
s34	32	18	8	19	254	181 (33)	142	441 (35)	13	21	18	16
s35	7	2	0	3	35	41 (13)	135	82 (22)	1	0	11	18
s36	3	2	6	17	178	189 (34)	110	159 (32)	3	4	0	4
s37	5	15	0	1	353	557 (36)	2	2 (1)	6	4	0	2
s38	11	8	0	0	194	119 (29)	53	29 (10)	17	11	11	11
s39	21	32	2	0	111	55 (22)	0	37 (11)	1	3	2	6
s40	1	2	1	0	145	206 (33)	11	56 (16)	31	26	3	7
s41	0	2	4	9	107	101 (28)	137	265 (31)	5	6	2	2
s42	0	0	9	10	121	191 (31)	35	62 (19)	10	15	10	2
s43	0	0	9	7	148	290 (35)	11	13 (7)	2	13	0	0
s44	3	7	3	13	293	392 (35)	32	76 (25)	10	7	7	0
s45	27	13	0	0	160	270 (34)	19	52 (19)	1	4	1	0
s46	0	1	6	11	226	307 (35)	52	175 (34)	1	4	2	4
s47	3	5	2	1	261	438 (36)	159	319 (36)	7	6	1	5
s48	6	1	0	2	25	52 (18)	41	35 (13)	2	5	20	12
s49	3	10	6	8	104	54 (15)	54	22 (7)	14	8	1	1
s50	23	48	4	9	48	229 (24)	2	0 (0)	0	0	5	6
s51	9	9	3	1	354	170 (31)	136	173 (29)	0	3	3	1
s52	0	0	2	1	20	74 (23)	57	139 (31)	3	8	0	2
s53	2	2	0	2	370	176 (30)	16	25 (8)	3	3	1	4
s54	3	14	1	0	149	107 (18)	66	51 (17)	7	6	7	4
Mean	6.11	9.33	3.43	3.81	152	178 (25.8)	87.0	116 (21.1)	5.82	7.31	4.54	5.30
Std	9.27	13.2	3.54	4.63	115	136 (8.99)	79.6	104 (10.1)	6.97	8.55	5.39	5.86

*The total number of epochs in training and testing phases of the MI and SSVEP BCI paradigms is 100, respectively. The total number of epochs in training or testing phases of the ERP paradigm is 1,980 or 2,160. Numbers in parentheses indicate that the number of target 36 characters including spaces in “PATTERN_RECOGNITION_MACHINE_LEARNING” have been affected by artifact-contaminated epochs. When the number is 36, all copying processes contained the effect of artifacts*.

**Figure 2:**
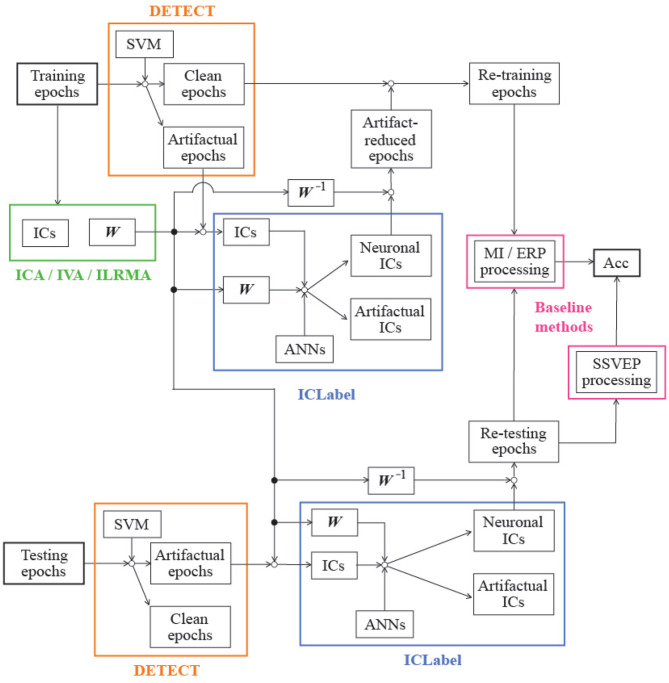
Block diagram of assessment procedure.

Through the epoch identification process, artifact-contaminated epochs in both the training and the testing phases were detected. In the training phase, we also assumed that artifact-reduced epochs contribute to the construction of an effective classifier if the artifact reduction technique effectively reduced artifactual effects from the artifact-contaminated epochs. Therefore, the demixing matrix ***W*** was first trained by using all epochs in the training phase, and artifactual ICs were then removed from the artifact-contaminated epochs using the artifact reduction process described in sections 2.2, 2.3, and 2.4. After artifact reduction, the clean and artifact-reduced epochs in the training phase were applied to the baseline analysis methods described in section 3. For performance evaluation, the artifact-contaminated epochs in the testing phase were used to compute the classification accuracy of the artifact-reduced epochs:

(31) $$\begin{array}{r} Acc=\frac{N_{\text{correct}}}{N_{\text{total}}}\times 100\%, \end{array}$$

where *N*_correct_ is the number of correct predictions, and *N*_total_ is the total number of artifact-contaminated epochs in the testing phase, as listed in Table 1, when the BCI paradigm was MI or SSVEP. Note that ERP data requires an averaging process for finding obvious feature waveforms (e.g., N200 and P300), and the averaged waveform relates to the classification performance. In other words, we cannot calculate the classification accuracy for each artifact-contaminated epoch in the paradigm. The numbers in parentheses in Table 1 indicate the number of characters affected by artifacts (*N*_total_), which is directly related to the assessment results. Figure 2 shows the block diagram of the assessment procedure used in this study.

A three-way repeated measures analysis of variances (ANOVAs) was applied to the classification accuracy to explore the effect of the two sessions, three BCI paradigms, and three artifact reduction methods. In addition, artifact-reduced signals obtained using ICA, IVA, and ILRMA and that were reconstructed from muscular or ocular artifact-contaminated signals were visualized to qualitatively investigate the artifact reduction performances.

## 5. Results

### 5.1. BCI Performance Before/After Applying Artifact Reduction Technique

**Figure 3:**
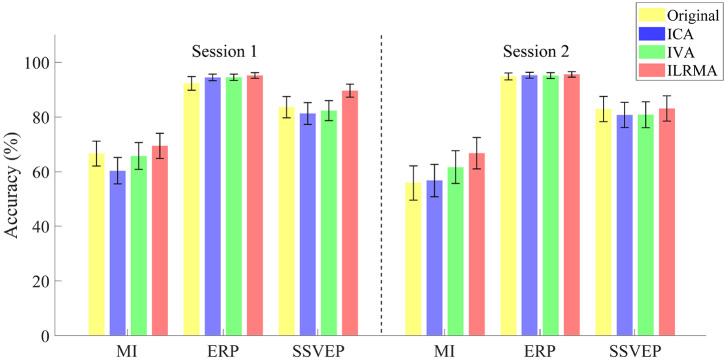
Averaged classification accuracies over all subjects for sessions, paradigms, and artifact reduction methods. Original indicates the results of using all data without artifact reduction method.

Table 2 lists the classification accuracies for all subjects, sessions, paradigms, and artifact reduction methods. In addition, Figure 3 shows the averaged classification accuracies over all subjects. A three-way repeated measures ANOVA using the classification accuracies of all subjects showed the significant main effects of the BCI paradigms [*F*_(2, 829)_ = 113.09, *p* < 0.001] and artifact reduction methods [*F*_(2, 829)_ = 3.05, *p* = 0.048]; however, it did not show any significant main effect of the sessions [*F*_(1, 829)_ = 1.29, *p* = 0.256]. There were no interaction effects among them. *post-hoc* analysis using Tukey test revealed that the ICA and ILRMA results had a significant difference (*p* = 0.039).

**Table 2:** Classification accuracies for all subjects, sessions, paradigms, and artifact reduction methods.

**Subject**	**ICA**						**IVA**						**ILRMA**					
	**MI**		**ERP**		**SSVEP**		**MI**		**ERP**		**SSVEP**		**MI**		**ERP**		**SSVEP**	
	**Se1**	**Se2**	**Se1**	**Se2**	**Se1**	**Se2**	**Se1**	**Se2**	**Se1**	**Se2**	**Se1**	**Se2**	**Se1**	**Se2**	**Se1**	**Se2**	**Se1**	**Se2**
s1	0	33.3	55.6	**90.9**	**100**	100	0	16.7	**61.1**	81.8	50.0	100	0	**66.7**	**61.1**	**90.9**	**100**	100
s2	–	0	100	100	–	–	–	**100**	100	100	–	–	–	**100**	100	100	–	–
s3	–	100	100	100	80.0	50.0	–	100	100	100	**90.0**	**100**	–	100	100	100	**90.0**	**100**
s4	–	–	85.7	100	100	–	–	–	85.7	100	100	–	–	–	85.7	100	100	–
s5	100	80.0	–	56.0	**90.9**	100	100	80.0	–	56.0	72.7	100	100	80.0	–	56.0	**90.9**	100
s6	100	66.7	100	100	92.3	100	100	66.7	100	100	92.3	100	100	66.7	100	100	**96.2**	100
s7	**50.0**	62.5	100	100	85.7	100	46.7	62.5	100	100	85.7	100	**50.0**	62.5	100	100	85.7	100
s8	–	100	81.3	**100**	50.0	**40.0**	–	100	**87.5**	95.5	50.0	20.0	–	100	**87.5**	**100**	50.0	**40.0**
s9	–	100	100	100	55.6	0	–	100	100	100	55.6	0	–	100	100	100	55.6	0
s10	0	–	100	100	–	100	**50.0**	–	100	100	–	100	**50.0**	–	100	100	–	100
s11	75.0	–	100	100	–	–	**100**	–	100	100	–	–	**100**	–	100	100	–	–
s12	37.5	50.0	100	96.8	0	100	43.8	50.0	100	96.8	0	100	**50.0**	50.0	100	96.8	**100**	100
s13	–	0	100	100	100	0	–	0	100	100	100	0	–	0	100	100	100	0
s14	–	0	100	96.9	83.3	81.8	–	0	100	96.9	83.3	81.8	–	0	100	96.9	83.3	**90.9**
s15	70.0	0	100	100	100	87.5	70.0	0	100	100	100	87.5	70.0	0	100	100	100	**93.8**
s16	0	70.0	100	100	–	100	**100**	**90.0**	100	100	–	100	**100**	**90.0**	100	100	–	100
s17	86.1	**50.0**	**93.3**	91.2	0	81.0	86.1	33.3	90.0	91.2	**100**	81.0	86.1	**50.0**	**93.3**	**94.1**	**100**	**85.7**
s18	**81.8**	88.9	100	100	100	100	72.7	88.9	100	100	100	100	**81.8**	88.9	100	100	100	100
s19	100	71.4	100	100	73.3	100	100	**85.7**	100	100	73.3	100	100	**85.7**	100	100	**80.0**	100
s20	**71.4**	0	100	100	100	100	57.1	0	100	100	100	100	**71.4**	0	100	100	100	100
s21	86.7	–	100	100	**100**	100	93.3	–	100	100	88.9	100	**96.7**	–	100	100	**100**	100
s22	75.0	–	93.8	88.9	100	0	**87.5**	–	93.8	88.9	100	0	**87.5**	–	93.8	88.9	100	0
s23	–	–	**86.2**	69.2	47.2	0	–	–	72.4	**73.1**	52.8	0	–	–	**86.2**	**73.1**	**55.6**	0
s24	0	25.0	100	90.3	0	80.0	0	**75.0**	100	**93.6**	0	80.0	0	**75.0**	100	**93.6**	**100**	80.0
s25	27.3	**50.0**	100	93.3	100	75.0	**45.5**	0	100	93.3	100	**100**	**45.5**	**50.0**	100	93.3	100	**100**
s26	100	–	**83.3**	87.5	90.9	100	100	–	80.6	87.5	90.9	100	100	–	**83.3**	**90.6**	90.9	100
s27	41.7	0	100	100	100	85.7	50.0	0	100	100	100	92.9	**58.3**	**100**	100	100	100	**100**
s28	83.3	–	100	94.7	100	100	**100**	–	100	94.7	100	100	**100**	–	100	94.7	100	100
s29	65.0	50.0	88.0	100	75.0	100	85.0	75.0	**96.0**	100	75.0	100	**100**	**100**	**96.0**	100	75.0	100
s30	55.6	–	82.4	92.9	100	–	55.6	–	82.4	92.9	100	–	55.6	–	**85.3**	92.9	100	–
s31	100	0	100	100	–	–	100	0	100	100	–	–	100	0	100	100	–	–
s32	43.3	100	100	100	88.2	95.2	44.8	100	100	100	**91.2**	95.2	**52.2**	100	100	100	**91.2**	95.2
s33	100	–	100	93.3	100	0	100	–	100	93.3	100	0	100	–	100	93.3	100	0
s34	55.6	42.1	87.8	91.4	76.2	81.3	55.6	**47.4**	**90.9**	**97.1**	76.2	81.3	55.6	**47.4**	**90.9**	**97.1**	**90.5**	81.3
s35	**50.0**	66.7	100	95.5	–	83.3	0	66.7	100	95.5	–	83.3	**50.0**	66.7	100	95.5	–	83.3
s36	50.0	94.1	100	87.5	100	100	50.0	94.1	100	87.5	100	100	50.0	94.1	100	87.5	100	100
s37	86.7	100	69.4	100	100	100	**100**	100	72.2	100	100	100	**100**	100	**75.0**	100	100	100
s38	**75.0**	–	96.6	100	100	72.7	62.5	–	96.6	100	100	72.7	**75.0**	–	96.6	100	100	72.7
s39	62.5	–	86.4	100	100	**83.3**	62.5	–	86.4	100	100	50.0	62.5	–	86.4	100	100	**83.3**
s40	0	–	97.0	100	**57.7**	85.7	0	–	90.9	100	46.2	85.7	0	–	97.0	100	**57.7**	85.7
s41	50.0	66.7	100	100	**83.3**	100	50.0	66.7	100	100	66.7	100	50.0	66.7	100	100	**83.3**	100
s42	–	80.0	100	94.7	66.7	100	–	80.0	100	94.7	**73.3**	100	–	80.0	100	94.7	**73.3**	100
s43	–	85.7	100	100	100	–	–	85.7	100	100	100	–	–	85.7	100	100	100	–
s44	100	100	100	100	0	–	100	100	100	100	**100**	–	100	100	100	100	**100**	–
s45	76.9	–	91.2	94.7	100	–	**92.3**	–	91.2	94.7	100	–	**92.3**	–	91.2	94.7	100	–
s46	100	63.6	100	94.1	100	100	100	63.6	100	94.1	100	100	100	63.6	100	94.1	100	100
s47	80.0	100	100	**100**	33.3	80.0	80.0	100	100	97.2	33.3	80.0	80.0	100	100	**100**	33.3	80.0
s48	100	0	100	100	100	100	100	0	100	100	100	100	100	0	100	100	100	100
s49	50.0	50.0	100	100	100	100	60.0	50.0	100	100	87.5	100	**70.0**	50.0	100	100	100	100
s50	60.4	55.6	87.5	–	–	83.3	62.5	44.4	87.5	–	–	83.3	**68.8**	**66.7**	**91.7**	–	–	83.3
s51	44.4	0	87.1	89.7	66.7	100	44.4	**100**	**90.3**	89.7	66.7	100	44.4	**100**	**90.3**	89.7	66.7	100
s52	–	100	100	100	75.0	100	–	100	100	100	**100**	100	–	100	100	100	**100**	100
s53	0	100	96.7	100	100	100	0	100	96.7	100	100	75.0	0	100	96.7	100	100	100
s54	57.1	–	100	100	83.3	100	42.9	–	100	100	**100**	100	57.1	–	100	100	**100**	100
Mean	61.6	59.4	95.3	96.0	82.0	81.4	66.3	62.1	95.3	96.0	83.0	81.5	**70.0**	**67.3**	**96.0**	**96.4**	**90.4**	**83.8**
SE	4.91	5.98	1.22	1.08	4.01	4.66	4.93	6.09	1.17	1.06	3.70	4.78	4.65	5.80	1.04	1.03	2.40	4.65

*Hyphens indicate that no artifact-contaminated epochs existed in the testing phase on the session. Bold values indicate that the accuracies over the three artifact reduction methods have different values and the results were higher than the other ones*.

The classification accuracies obtained using ILRMA in all cases were always equal to or higher than the higher accuracy of using ICA or IVA (see Table 2). In particular, ILRMA improved the discriminability of artifact-reduced data for 31 subjects in session 1 and 24 subjects in session 2. When there was a difference in the artifact reduction performance, we highlighted the superior results in bold in the table. The averaged accuracy of using ILRMA in all BCI paradigms was also equal to or higher than that of using ICA and IVA (Figure 3). Interestingly, in some cases, artifact-contaminated data showed higher BCI performance than ICA and IVA. However, ILRMA always showed equal or higher performance compared to artifact-contaminated situations. For these results, ILRMA salvaged effective components for solving the classification problem from artifact-contaminated signals in the MI and SSVEP paradigms. Conversely, in the ERP paradigm, ICA was sufficient to remove the artifactual components and achieved almost 100% accuracy.

### 5.2. Representation of Original and Artifact-Reduced Signals

**Figure 4:**
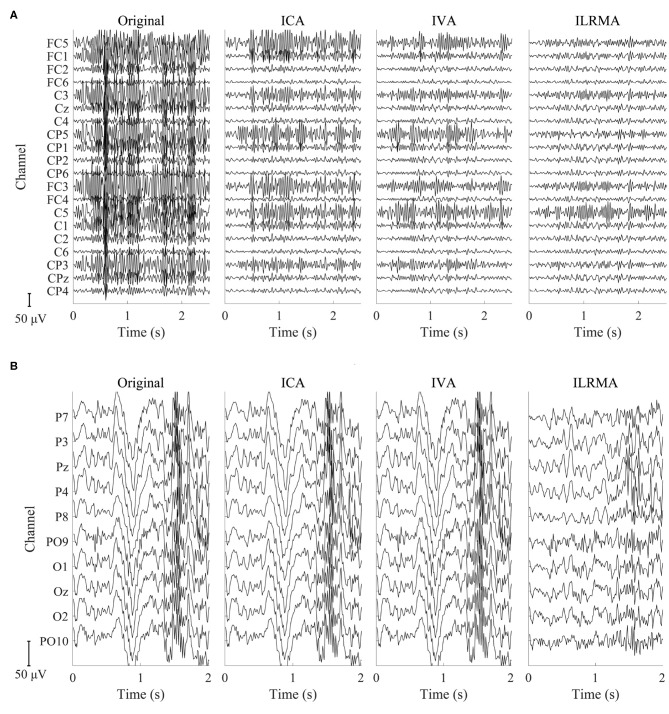
Artifact-contaminated EEG epoch and artifact-reduced EEG epochs estimated by ICA, IVA, and ILRMA in the **(A)** MI paradigm and **(B)** SSVEP paradigm.

**Figure 5:**
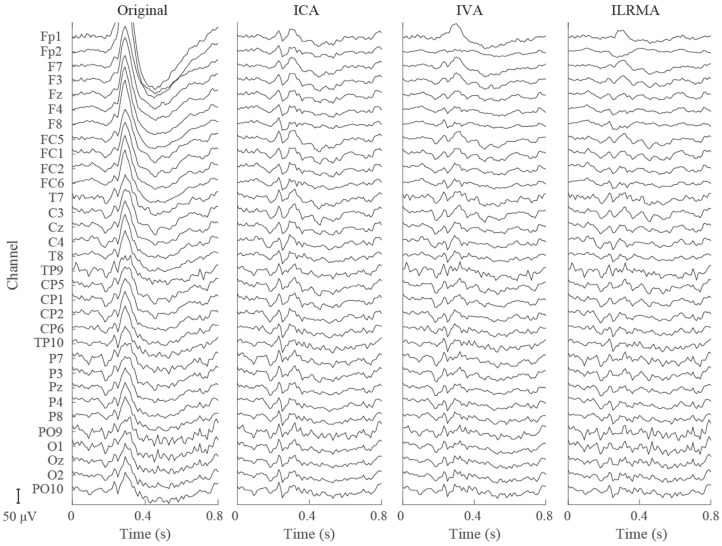
Artifact-contaminated EEG epoch and artifact-reduced EEG epochs estimated by ICA, IVA, and ILRMA in the ERP paradigm.

Figures 4, 5 show artifact-contaminated EEG epochs and artifact-reduced EEG epochs obtained using ICA, IVA, and ILRMA in the MI, ERP, and SSVEP paradigms. They were qualitatively indicated that ILRMA could better remove artifact effects compared to ICA and IVA. In addition, the task-independent components were removed by ILRMA to leave characteristic features in each paradigm (e.g., event-related desynchronization caused by motor imagery and evoked potential by steady-state visual stimulus) instead of the attenuating power of all frequency components. This resulted in improvements in these BCI performances.

## 6. Discussion

### 6.1. Automatic Processing Architecture

ICA-based artifact reduction techniques have been widely used in the field of EEG signal processing because of their powerful signal separation accuracy, simplicity (low computational cost), and ease of use (Delorme et al., 2007; Dimigen, 2019; Jiang et al., 2019). The techniques for limiting ocular and muscular artifacts (Chen et al., 2019; Tian et al., 2020) other than the ICA family are useful if they are integrated in a cascade-type processing module, which can automatically identify the type of artifact contained in the EEG observation. A simple filtering (linear combination) approach such as ICA, which multiplies the demixing matrix ***W*** as a filter, is faster and user-friendly. IVA and ILRMA use this property and can sufficiently cope with online processing as long as they can learn the demixing matrix. In addition, these algorithms can benefit from the ICLabel classifier (Pion-Tonachini et al., 2019) for IC identification to realize an automatic artifact reduction method. Thus, the ILRMA-based artifact reduction technique (1) has higher accuracy than ICA, (2) has low computational cost (equivalent to ICA) in an online process, (3) is a changeable module for the ICA decomposition function, and (4) can simultaneously remove multiple types of artifacts. Note that the ICLabel classifier is expected to be updated frequently in the future. Although the EEGLAB toolbox keeps track of updates in the run_ICL function, the label assignment results may change depending on the updates. In this study, we selected the “default” version for IC identification.

### 6.2. Efficacy of Artifact Reduction for BCIs

Researchers who propose original artifact reduction techniques for BCIs should describe not only the signal quality but also the discriminative performance of the extracted components to demonstrate their efficacy in BCIs. The performances of proposed artifact reduction techniques in most previous studies were evaluated and ranked based on a metric (e.g., signal-to-noise ratio and correlation) that indicates how the signal quality of the estimated neuronal sources was preserved (Islam et al., 2016). We do not know the original (true) neuronal sources of EEG observations; thus, synthetic data whose pseudo-neuronal/pseudo-artifactual sources and mixing process are known were usually used to calculate the metric (Chen et al., 2019; Mucarquer et al., 2019). After the quantified evaluation of signal quality in the estimated sources through the proposed artifact reduction technique, the separation ability for real data is qualitatively shown (Blum et al., 2019; Kanoga et al., 2019a). However, the evaluation of the discriminative performance of the remaining sources (extracted components) is not a major/standard quantitative one in this field. To implement an artifact reduction technique into the BCI framework, it is more important to know which aspect of the technique is the most crucial to the classification/identification performance. In this study, we demonstrated improved MI- and SSVEP-based BCI performances using our proposed technique, which represents common and recurrent properties of artifactual waveforms into trials over all classes in low-rank bases and automatically removes them. Except for session 2 in the MI-based BCI performance, our proposed method showed over 70% averaged accuracies (Table 2), which is required for satisfactory BCI control (Sellers et al., 2006). When we consider the time latency during an MI period and change the starting time point of MI features from fixed to flexible by using time window selection algorithm such as correlation-based time window selection (Feng et al., 2018), the average performance of the MI-based BCI might be improved. Furthermore, using other kind of feature extraction method such as sparse FBCSP (Zhang et al., 2015) is one of good solution to improve the MI-based BCI performance because the method automatically chose the filter bands with superior accuracies compared with FBCSP. In the case of the ERP-based BCI, ICA was already effective enough. Therefore, the superiority of ILRMA could not be confirmed; however, its performance is equivalent to that of ICA. Although this paper did not present the quantitative signal quality of the estimated neuronal sources because we did not prepare synthetic data to avoid the artificial bias of neuronal characteristics (all EEG observations have unclear individual differences such as amplitudes and latencies, so we could not easily predict the features and generate pseudo/synthetic data), the classification/identification results obtained with three well-known BCI paradigms should be helpful information for practitioners and implementers.

### 6.3. Limitations

The results obtained using the DETECT toolbox were treated as the grand truth. However, the muscular label reflected the characteristics of “clenching” and “flexing both arms.” Other types of muscular artifacts, such as “changing head direction,” may not be extracted as artifactual epochs. In addition, the “100% accuracy” was sometimes calculated using only one testing epoch although other accuracies were calculated using more epochs (e.g., 20). The comparison of artifact reduction techniques was fair because the number of artifact-contaminated epochs was the same over the factor. However, the comparisons of BCI paradigms and multiday effects were not fair: each factor has different numbers of artifact-contaminated epochs. Evaluating the techniques as fairly as possible by using the DETECT toolbox is very difficult. For solving this problem, an artifact-contaminated EEG dataset with multiple types of intensity-manipulated artifacts is required in this research field to enable rapid developments in artifact reduction techniques for BCIs.

### 6.4. Future Works

Further improvement of ILRMA-based artifact reduction techniques is expected through the introduction of an identification algorithm for decomposed frequency components and a soft-threshold-like wavelet-enhanced ICA (Castellanos and Makarov, 2006). Despite the fact that ILRMA decomposes the STFT of the original signals up to each frequency bin, our automatic processing architecture reconstructs artifact-reduced signals by replacing artifactual source(s) with zeros (replacing entire frequency bins with zeros) to adapt the ICLabel classifier, which needs time-series ICs. In other words, a lot of neural information is lost in the reduction step. Signal reconstruction should be made more sophisticated by considering the effective frequency band adjusted to the BCI paradigm.

Moreover, we need further investigations of artifact reduction methods in practical situations such as using wearable devices that have small number of channels (in an extreme case, the number of channels is only one) for EEG measurements. In such situation, the performance of artifact reduction techniques will change and might be decreased. Recent studies attempt to propose a generic artifact removal algorithm (Chen et al., 2019). Unlike the time-domain algorithm, frequency-domain methods (i.e., IVA and ILRMA) can separate single-channel data if the differences in data-driven spectral basis functions can be learned well (Kanoga and Mitsukura, 2014; Kanoga et al., 2019a). Thus, we will investigate our proposed algorithm in practical situations and extend it as a generic and user-friendly algorithm for reducing artifacts from EEG data.

The ICA family, including IVA and ILRMA, represents EEG observations through linear combinations of sources based on a time-invariant demixing matrix; the trained demixing matrix may sometimes cause instability through inter-/intrasubject variabilities. By integrating with a transfer learning algorithm (Pan and Yang, 2009; Tan et al., 2018), relearning from the general filter (demixing matrix) to the user-specific filter according to the data while performing online processing could potentially reduce the variability and provide more convenient and practical BCIs.

## Data Availability Statement

Publicly available datasets were analyzed in this study. This data can be found here: http://gigadb.org/dataset/view/id/100542/File_page/1.

## Author Contributions

SK obtained the initial idea for this study. All authors contributed to the planning and design of the study. SK and TH analyzed the data and interpreted the results. SK compiled the first draft of the article. All authors participated in revisions to finalize the draft of the manuscript.

## Conflict of Interest

The authors declare that the research was conducted in the absence of any commercial or financial relationships that could be construed as a potential conflict of interest.
